# ﻿A new arboreal species of *Vitronura* Yosii, 1969 and a new record of *Yuukianura* Yosii, 1955 (Collembola, Neanuridae) from China, with notes on the feeding behavior of Neanurinae

**DOI:** 10.3897/zookeys.1245.150272

**Published:** 2025-07-15

**Authors:** Ji-Gang Jiang, Daoyuan Yu

**Affiliations:** 1 College of Life and Environmental Science, Hunan University of Arts and Science, Changde 415000, Hunan, China Hunan University of Arts and Science Changde China; 2 Soil Ecology Lab, College of Resources and Environmental Sciences, Nanjing Agricultural University, Nanjing 210095, China Nanjing Agricultural University Nanjing China

**Keywords:** Checklist, feeding preference, Lobellini, new species, springtails, *Vitronuracheni* sp. nov., *Yuukianuraszeptyckii*

## Abstract

A new species, *Vitronuracheni***sp. nov.** (Collembola: Neanuridae) is described from Yongzhou, Hunan Province, China. The new species is characterized by the combination of following characters: 2+2 uncolored big eyes; labral chaetotaxy as 0/2, 2; cephalic tubercle Fr with chaeta O; cephalic tubercles Dl, L and So fused; mandibles with four teeth; maxillae with two lamellae, the long one with a tiny tooth and the short one with pointed tip; labrum truncated; claw without inner tooth; and femur and tibiotarsus with a tenant chaeta. *Yuukianuraszeptyckii* Deharveng & Weiner, 1984 was reported from China for the first time. Observations of a cultured population of *Y.szeptyckii* revealed feeding preferences and behavior that differ from those previously reported in other members of Neanurinae. A checklist of *Yuukianura* species and distributions is also provided.

## ﻿Introduction

*Vitronura* was established by Yosii (1969) as a subgenus under *Neanura* MacGillivray, 1893, and was raised to generic level by Cassagnau (1983). This genus is mainly characterized by the presence of separate tubercles Fr and An on the dorsal side of the head (Smolis and Deharveng 2006). Till now, 25 valid species of *Vitronura* have been reported worldwide (Bellinger et al. 1996–2025; Jiang et al. 2025). In 2024, during an entomological survey in Mountain Jiuyi National Nature Preserve, Hunan, China, a tree-dwelling new species of *Vitronura* was discovered and described herein.

*Yuukianura* Yosii, 1955 was initially established for the species *Protanuraaphoruroides* Yosii, 1953, characterized by its slender body shape, weakly-developed tubercles, and modified mouthparts. To date, a total of 11 species of *Yuukianura* have been reported from humid habitats across Asia, the Pacific Islands, Australia, and Great Britain (Deharveng et al. 2017; Smolis 2017; Kasai et al. 2024). In this paper, a species originally described from the Korean Peninsula, i.e., *Yuukianuraszeptyckii* Deharveng & Weiner, 1984, is recorded for the first time from Nanjing, China. In addition, we provide a report on the feeding preferences and behavior of this species based on observations of a cultured population.

## ﻿Material and methods

Specimens were collected by sieving and preserved in alcohol. They were mounted on slides using Hoyer’s solution and then dried for two weeks in an oven at 50 °C. Illustrations were made using a Nikon DS-Ri2 camera mounted on a Nikon 80i phase-contrast microscope and prepared for publication with Adobe Photoshop CS2. The terminology and layout of the tables used in this paper follow Deharveng (1983), Deharveng and Weiner (1984), Smolis and Deharveng (2006), and Smolis (2008).

Additionally, *Yuukianuraszeptyckii* was cultured in the laboratory to observe its feeding behavior. To establish the microcosms, plastic containers with a height of 10 cm and a base area of 10×20 cm were used, filled with a 2 cm-thick layer of moistened peat soil as the substrate. A ventilation opening was created in the lid and covered with gauze. The containers were placed in a climate-controlled chamber with a constant temperature of 20±0.5 °C and 75%±5% humidity. Day and night cycles were set to 12 hours each, with daytime light intensity ranging from 300 to 800 lux. The feeding preference of the new species was tested with three types of food resources: fungi (Brewer’s yeast, *Saccharomycescerevisiae*), plant seeds (oat flakes, *Avenasativa*), and slime mold (*Physarumpolycephalum*).

### ﻿Abbreviations

General morphology:

**Abd.** abdomen

**Ant.** antenna

**Cx** coxa

**Fe** femur

**Scx2** subcoxa 2

**Ti** tibiotarsus

**Th.** thorax

**Tr** trochanter

**VT** ventral tube

Groups of chaetae:

**Ag** antegenital

**An** anal

**Fu** furcal

**Vc** ventrocentral

**Ve** ventroexternal

**Vi** ventrointernal

**Vl** ventrolateral

**Vei** ventroexternointernales

**Vec** ventroexternocentrales

**Vel** ventroexternolaterales

**Vea** ventroexternoanterior

**Vem** ventroexternomedial

**Vep** ventroexternoposterior

Tubercles:

**An** antennal

**Fr** frontal

**Cl** clypeal

**De** dorsoexternal

**Di** dorsointernal

**Dl** dorsolateral

**L** lateral

**Oc** ocular

**So** subocular

Types of chaetae:

**Ml** long macrochaeta

**Mc** short macrochaeta

**Mcc** very short macrochaeta

**me** mesochaeta

**ms** s-microchaeta

**S** or **s** chaeta s

**Or** organite of ant. IV

**i** ordinary micro- or mesochaeta on ant. IV

**mou** cylindrical sensilla on ant. IV (“soies mousses” in Deharveng 1981)

**x** labial papilla x

**L**’ ordinary chaeta on abd. V

## ﻿Results

### ﻿Family Neanuridae Börner, 1901 sensu Yosii 1956

**Subfamily Neanurinae Börner, 1901**

**Tribe Lobellini Cassagnau, 1983**

#### ﻿Genus *Vitronura* Yosii, 1969

##### ﻿ Vitronura cheni sp. nov.

Taxon classificationAnimaliaPoduromorphaNeanuridae

066D6EC2-2C06-53E5-9E89-97DA6ED85952

https://zoobank.org/ACD8D18C-AEE9-4869-940E-B88218FB07DE

Figures 1–2 , 3, 4 , 5–8 , 9 , 10 , 11, 12 , 13 , 14 , Tables 1 , 2

###### Type material.

***Holotype***: • Male, Mountain Jiuyi National Nature Preserve, Ningyuan County, Yongzhou, Hunan Province, China, coordinates: 25.2538°N, 112.0188°E, alt. 1445 m, on trunk of pine trees, leg. Ji-Gang Jiang, 2.v.2024 (No. 2024050102). ***Paratypes***: • three females and one male, same data as the holotype. Type materials are deposited at the Key Laboratory of Zoology, Hunan University of Arts and Science (HUAS), Changde, Hunan Province, China.

###### Etymology.

The name of the species is dedicated to Professor Jian-Xiu Chen, Nanjing University, China, for his outstanding contributions to Chinese Collembola.

###### Diagnosis.

Two eyes per side on head, colorless; cephalic chaeta O present; body tubercles well differentiated; cephalic tubercles Dl, L and So fused; mandible with a basal tooth, a middle tooth and two apical teeth; maxilla with two lamellae, the longer one with one tiny tooth, and the shorter one needle-like; body dorsum macrochaetae usually slightly clavate; labrum truncated; claw without inner tooth; femur and tibiotarsus with a tenant chaeta.

###### Description.

**Figures 1, 2.**
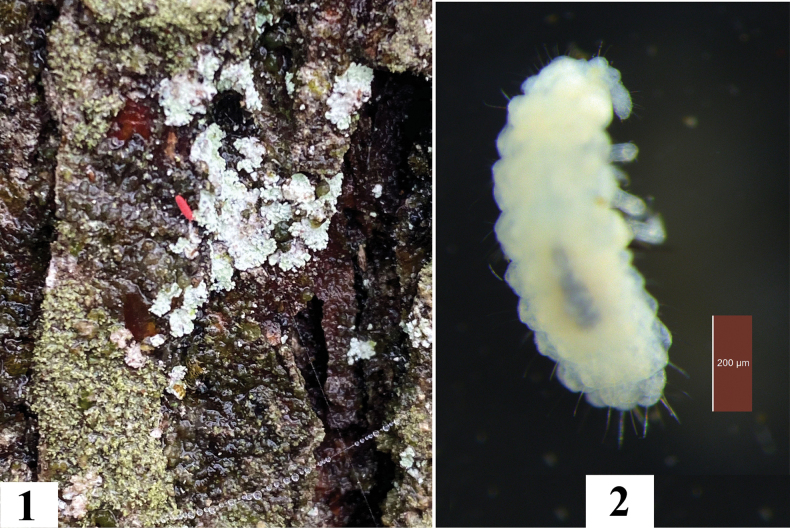
*Vitronuracheni* sp. nov.: **1.** Live specimen on pine tree; **2.** Preserved specimen in alcohol.

Body length: 1.4–1.7 mm. Color: living specimens red (Fig. 1) and whitish in alcohol (Fig. 2).

**Figures 3, 4.**
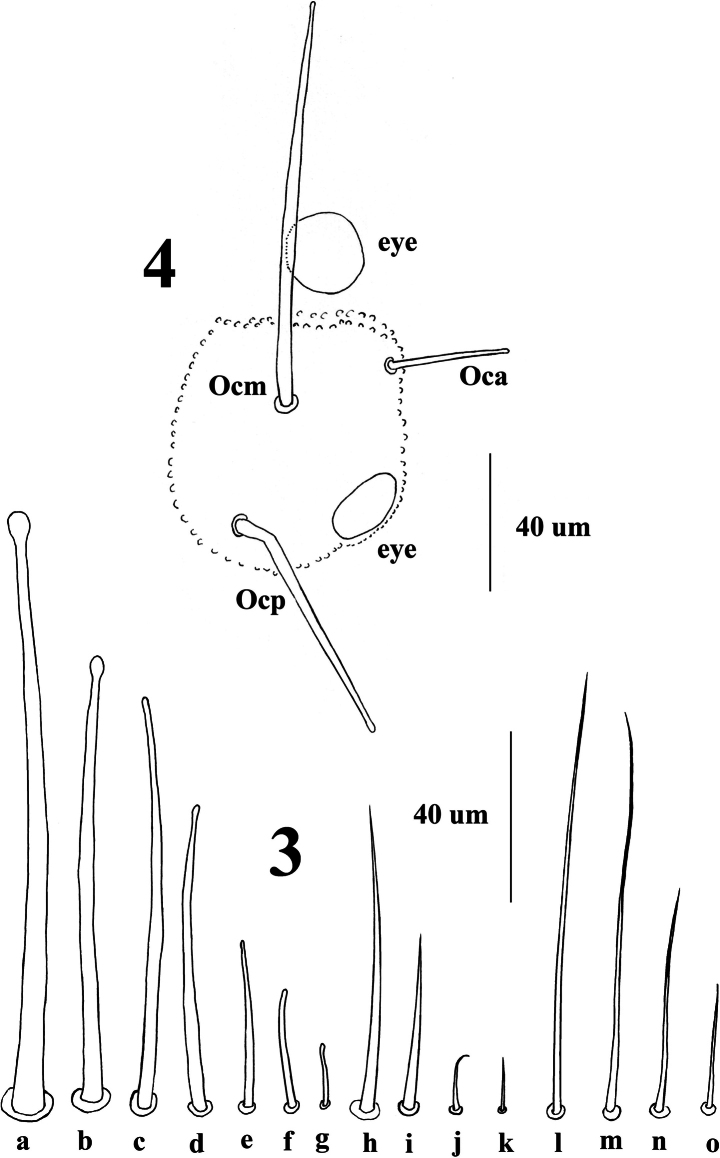
*Vitronuracheni* sp. nov.: **3.** Chaetal types: Ml (**a**), Mc (**b–d**), Mcc (**e–g**), me (**h–k**), tenent chaeta (**l–m**), s (**n**: chaeta s on Abd. V, **o**: chaeta s on Abd.I); **4.** Tubercle Oc (right side).

***Chaetal morphology*.** Dorsal ordinary chaetae of four types. Long macrochaetae (Ml) smooth, clavate (Fig. 3a). Short macrochaetae (Mc) shorter than Ml, clavate or with blunt tip and with variety of lengths (Fig. 3b, c, d). Very short macrochaetae (Mcc) morphologically similar to but shorter than Mc, with variety of lengths (Fig. 3e, f, g). Mesochaetae (me) smooth, pointed and with variable lengths (Fig. 3h, i, j, k). S-chaetae (s) on terga thin, smooth, usually shorter than Mc and equal to that of medium me, chaetae s on abd. V (Fig. 3n) longer than that of Abd. I–III (Fig. 3o). Tenent chaeta on tibiotarsus morphologically similar to but much longer than chaeta s, subequal to the longest Mc (Fig. 3l, m).

***Head*.** Each side with 2 uncolored big eyes, one anterior, but not included in Oc tubercle, the other one on the posterior part of Oc tubercle (Fig. 4).

**Figures 5–8.**
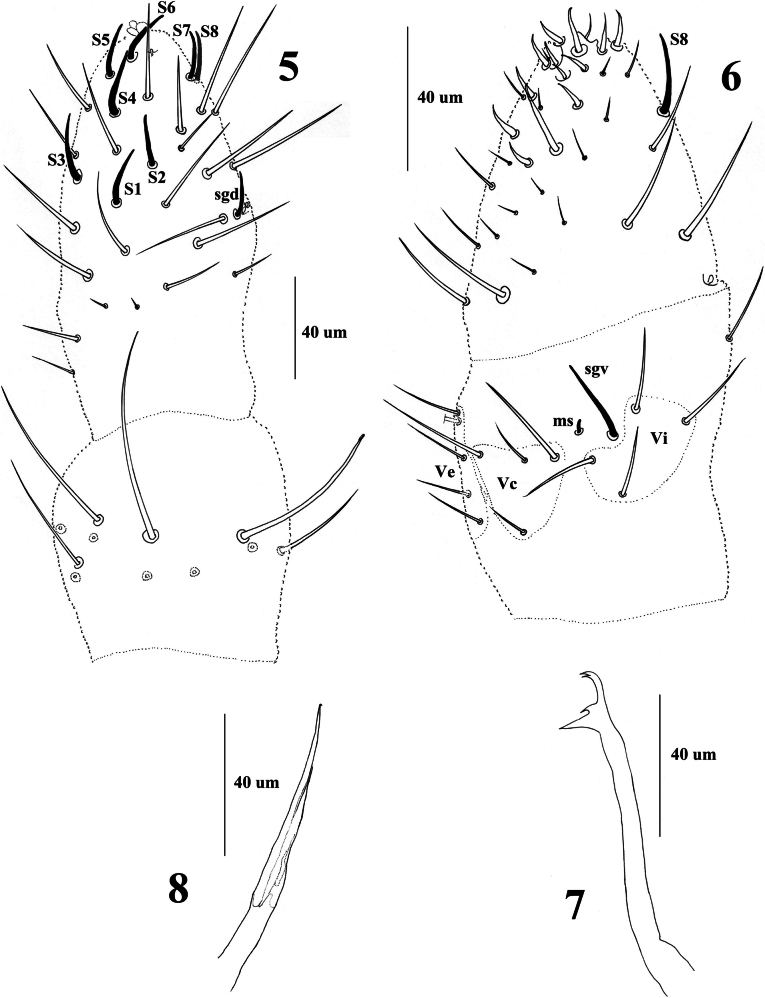
*Vitronuracheni* sp. nov.: **5.**Ant. II–IV (dorsal side); **6.**Ant. III–IV (ventral side); **7.** Mandible; **8.** Maxilla.

Antenna four-segmented. Ant. I with 7 chaetae. Ant. II with 11 chaetae. Ant. III dorsally fused to Ant. IV. Guard chaeta sgd of Ant. III not migrated distally, at the same level as the two sensory rods. Ant. IV dorsally with eight subequal, slightly thickened and blunt sensilla (S1–8), apically with three bulbs, subapical organite (or) present (Fig. 5). Ventral side of Ant. IV with about 14 pointed thickened chaetae (Fig. 6). On ventral side of Ant. III, Vi, Vc and Ve with 4, 4, 5 chaetae respectively (Fig. 6).

Mandible consisting of one basal tooth, one middle tooth and two apical teeth (Fig. 7). Maxilla consisting of two lamellae, the long lamella apically with a tiny tooth, and the short one with pointed tip (Fig. 8). Labrum truncated, labral formula as 0/2, 2. Labium with 11 chaetae and two papillae x.

**Figure 9.**
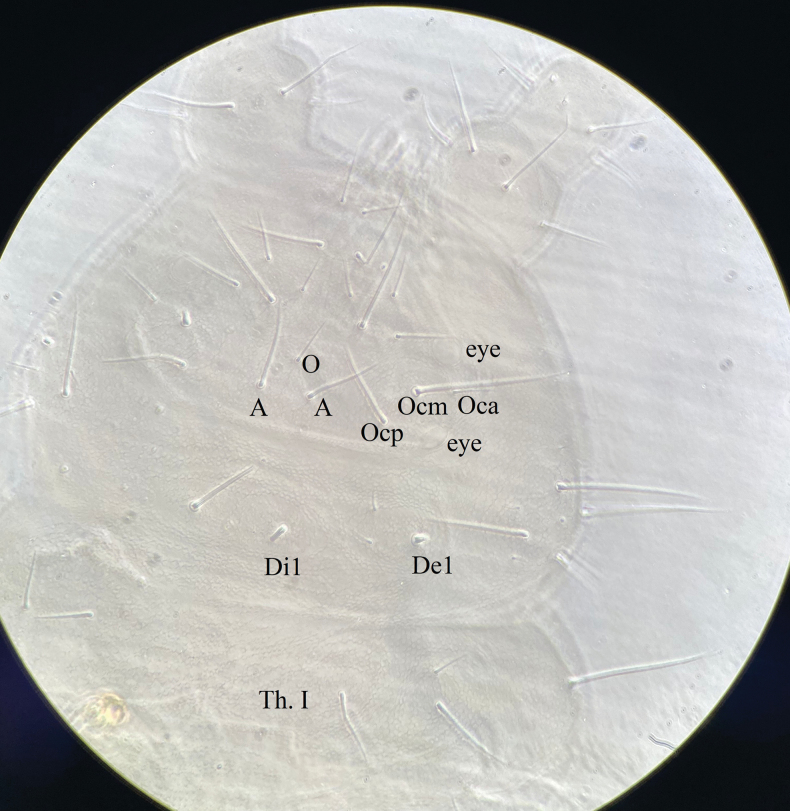
*Vitronuracheni* sp. nov.: tubercles and chaetotaxy on dorsal head.

Cephalic dorsal tubercles and chaetotaxy as in Table 1 and Fig. 9. Central area of head with six tubercles: one Cl, one Fr, two Oc and two An independent respectively; chaeta O present on tubercle Fr as a Mc. Dorso-posterior area with four separate tubercles: two Di and two De, chaeta Di2 and De2 as mes on tubercle De. Tubercles Dl, L and So on dorsal lateral area fused, with 11–12 chaetae.

**Table 1.** Cephalic tubercles and chaetotaxy of *V.cheni* sp. nov.

Tubercle or group of chaetae	Number and type of chaetae	Chaetae names
Cl	2 Mc	F
	2 Mc	G
An	1 Ml	B
Fr	3 Mc	C, D, E
	3 Mc	2A, O
Oc	1 Ml	Ocm
	1 Mc	Ocp
	1 mcc	Oca
Di	1 Mc	Di1
De	1 Ml	De1
	2 Mcc	Di2, De2
Dl+L+So	4 Ml+7–8 Mc (or me)	Uncertain

Ventral chaetotaxy of head. Group Vi with six chaetae, groups Vea with four, Vem with three and Vep with three chaetae, respectively.

**Figure 10.**
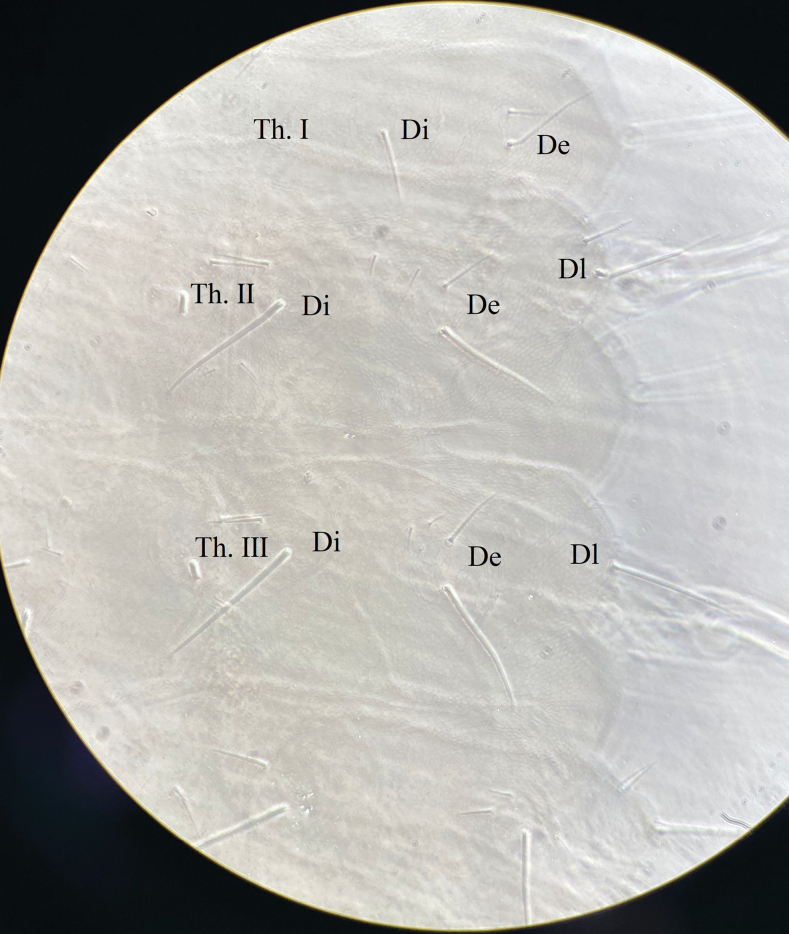
*Vitronuracheni* sp. nov.: tubercles and chaetotaxy on dorsal thorax.

**Figures 11, 12.**
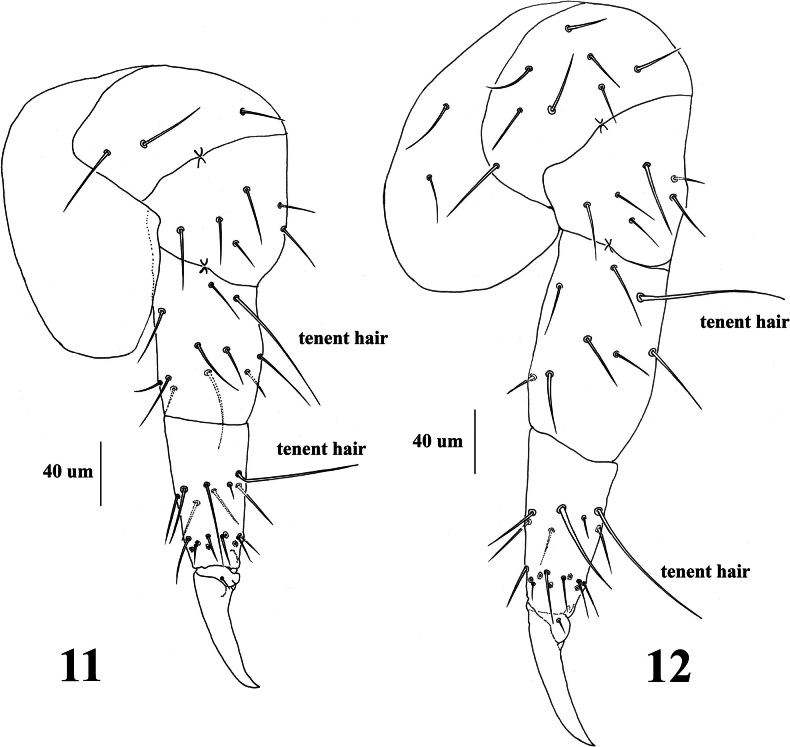
*Vitronuracheni* sp. nov.: **11.** Fore leg; **12.** Hind leg.

***Thorax*** (Fig. 10 and Table 2). Th. I with 3+3 tubercles, Di with 1, De with 2, Dl with 1 chaeta. Th. II with 4+4 tubercles. Di with three chaetae. De with five (4+s) or four (3+s) chaetae. Dl with five (3+s+ms) chaetae. Tubercle L with three chaetae. Th. III with 4+4 tubercles, Di with three chaetae, De with five (4+s) chaetae, Dl with four (3+s) chaetae, and L with three chaetae. Chaetotaxy of thorax and legs listed in Table 2. Unguis without inner or lateral teeth; unguiculus absent. Each Femur and tibiotarsus respectively with a tenent chaeta on all legs (Figures 3l, m, 11, 12).

**Table 2.** Postcephalic dorsal tubercles and chaetotaxy of *V.cheni* sp. nov.

Terga					Legs				
	Di	De	Dl	L	Scx2	Cx	Tr	Fe	T
Th. I	Mc	Mc+Mcc	Ml	–	0	3	6	11	19
Th. II	Ml+Mc+Mcc (or me	Ml+3Mc+ +s	Ml+Mc+ Mcc+s+ms	Ml+2Mcc	2	7	6	8	19
Th. III	Ml+Mc+ Mcc (or me	Ml+3Mc+ +s	Ml+Mc+ Mcc +s	Ml+2Mcc	2	8	6	8	18
Terga					Sterna				
Abd. I	Ml+Mc	Ml+ 2Mcc+s	Ml+Mcc	Ml+2Mcc	VT: 4				
Abd. II	Ml+ Mc	Ml+ 2Mcc+s	Ml+Mcc	Ml+2Mcc	Vi: 1, Ve: 4, Ve1: 0				
Abd. III	Ml+ Mc	Ml+ 2Mcc+s	Ml+Mcc	Ml+2Mcc	Fu: 4, Ve: 4				
Abd. IV	Ml+ Mcc	Ml+Mcc+s	Ml+Mc +Mcc	Ml+2Mc +3me	Vei: 2, Vec: 3, Vel: 4, Vl: 5				
Abd. V	Ml+2Mcc	s+2Ml+Mc+Mcc		4me	Ag: 3, Vl: 0				
Abd. VI	4Ml+3me				Ve: 13, An: 3 mi				

**Figure 13.**
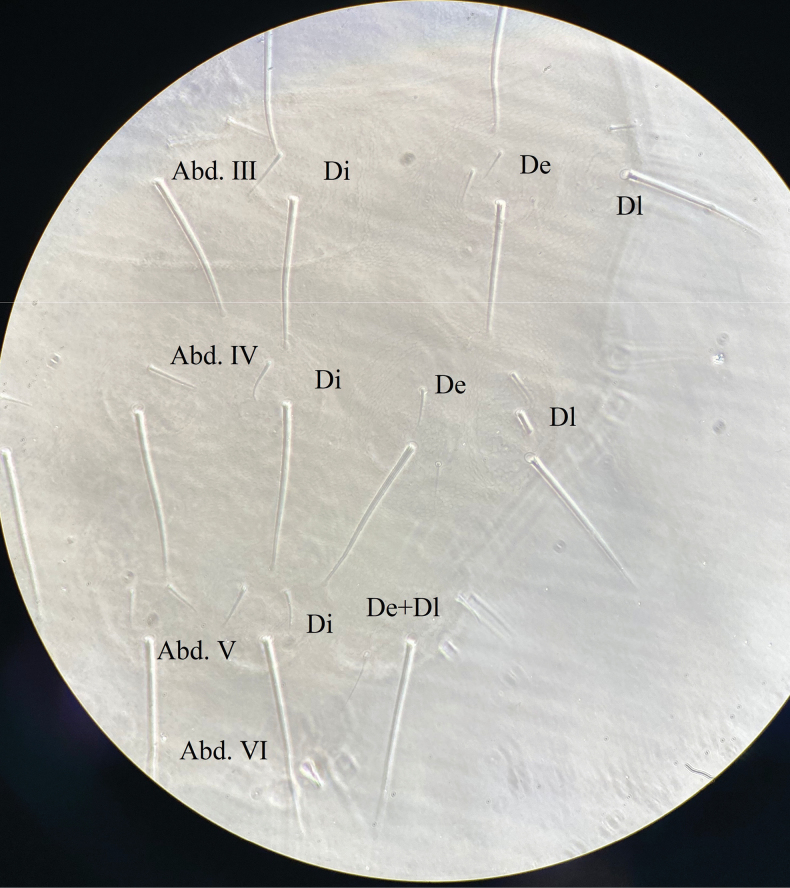
*Vitronuracheni* sp. nov.: tubercles and chaetotaxy on dorsal side of Abd. III –VI.

**Figure 14.**
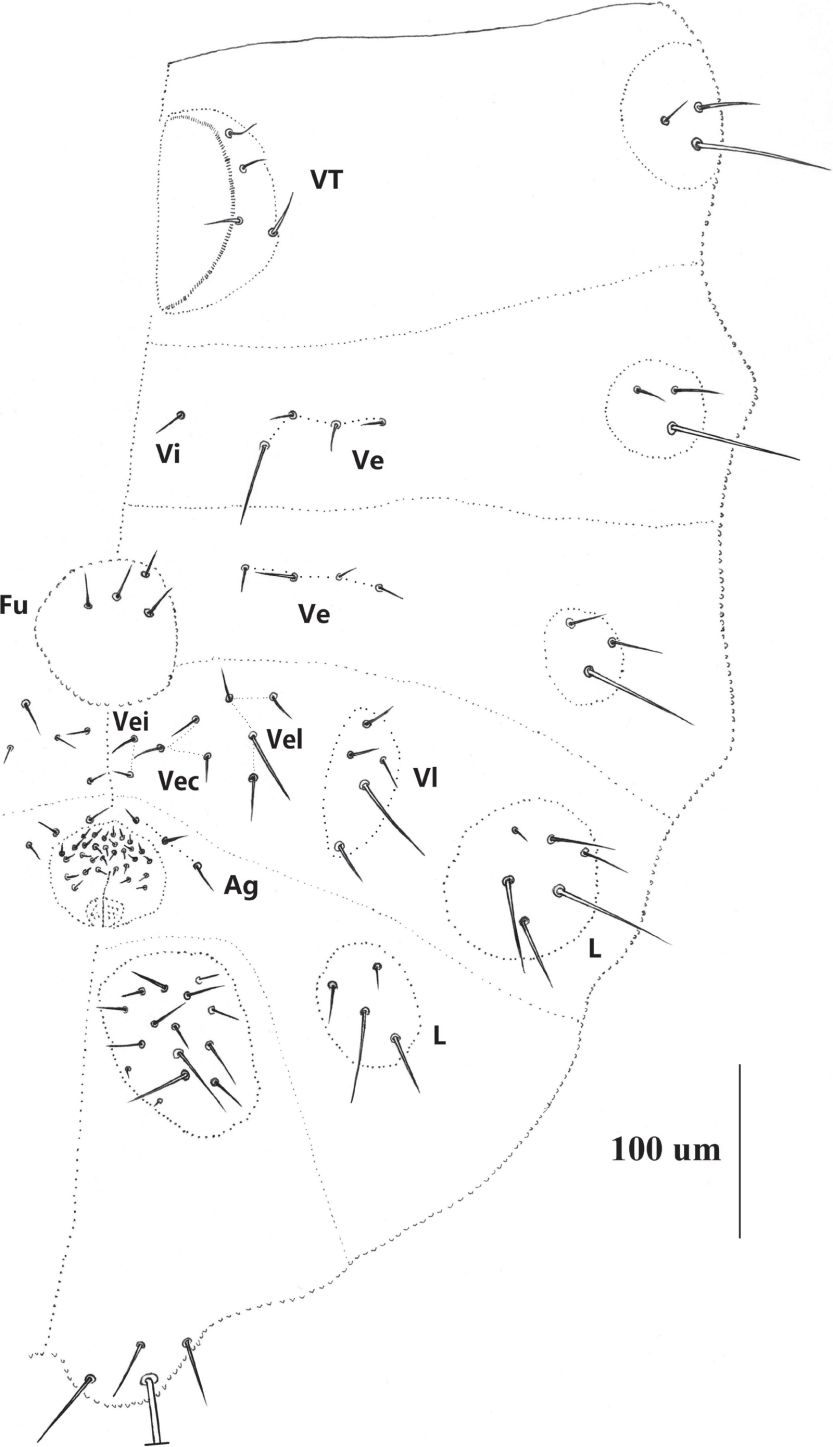
*Vitronuracheni* sp. nov.: ventral side of Abd. I–VI.

***Abdomen*** (Figs 13, 14 and Table 2). Abd. I–III with 4+4 tubercles each, Di with two, De with four (3+s), Dl with two and L with 3 chaetae, respectively. Abd. IV with 4+4 tubercles, Di with two, De with three (2+s), Dl with three and L with 6 chaetae, respectively. Abd. V dorsally with 2+2 tubercles, two Di separated, each with three chaetae, tubercle De with only one chaeta s, and fused to tubercle Dl, Dl with four chaetae. Abd. VI with 1+1 tubercles, and seven chaetae on each tubercle. VT with 4+4 chaetae (Fig. 14). Furcular remnant with 4 chaetae (Fig. 14).

###### Ecology.

The new species was found on the trunk of pine trees.

###### Remarks on taxonomy.

Morphologically, *Vitronuracheni* sp. nov. strongly resembles *V.tianmua* Jiang & Xia, 2018 from Zhejiang Province, China, in the presence of 3 chaetae on cephalic tubercle Oc, the presence of 3 chaetae on cephalic tubercle De, the presence of 4 teeth on the mandible, no inner tooth on the claw and no ogival labium. However, the new species can be distinguished from the latter by the following features: shape of body chaetae smooth and clavate in the new species versus blunt and serrate in the latter; the presence of chaeta O on cephalic tubercle Fr or not (chaeta O present in the new species versus chaeta O absent in the latter).

The new species is close to *V.macgillivrayi* (Denis, 1933) from Costa Rica in the following characters: 4 teeth on mandible, smooth body chaetae, tubercle Fr with chaeta O, 3 chaetae on tubercle Oc, and no inner tooth on claw. However, *Vitronuracheni* sp. nov. can be differentiated from *V.macgillivrayi* by the cephalic tubercle Di separate instead of the cephalic tubercle Di fused in the latter.

*Vitronuracheni* sp. nov. is similar to *Vitronuraparaacuta* Wang, Wang & Jiang, 2016 from Hunan Province, China by sharing the following characters: 4 teeth on mandible, body macrochaetae smooth and clavate, tubercle Fr with 3 chaetae, tubercle Oc with 3 chaetae, tubercle De + Dl with 5 (s+4) chaetae. However, *Vitronuracheni* sp. nov. can be separated from *Vitronuraparaacuta* by having a truncate labrum, no inner tooth on the claw versus having an ogival labrum and an inner tooth on the claw in the latter. The following combination of characters of the new species can differentiate it from all known *Vitronura* species: big eyes on the head, an outstanding tenant chaeta on the tibiotarsus, 4 teeth on the mandible, and no inner tooth on the claw.

###### Remarks on ecology.

Up to now, 25 valid species of the genus *Vitronura* Yosii, 1969 have been reported worldwide (Jiang et al. 2025), living in soil or under decayed leaves on the ground. By contrast, during our survey at 23 different locations in the ranges of Mt. Jiuyi, *Vitronuracheni* sp. nov. was collected exclusively from the trunks of pine trees, but was absent from all ground litter and soil samples, indicating that this species may have different environmental and resource preferences from other *Vitronura* species. Potential morphological adaptations for aboreal life include the large eyes and tenent chaetae on the femur and tibiotarsus, which may facilitate its locomotion between tree barks.

### ﻿New record

#### ﻿Genus *Yuukianura* Yosii, 1955

##### ﻿ Yuukianura szeptyckii

Taxon classificationAnimaliaPoduromorphaNeanuridae

Deharveng & Weiner, 1984

C88B9812-830E-5C91-8E9A-ADFA491A7937

**Figure 15.**
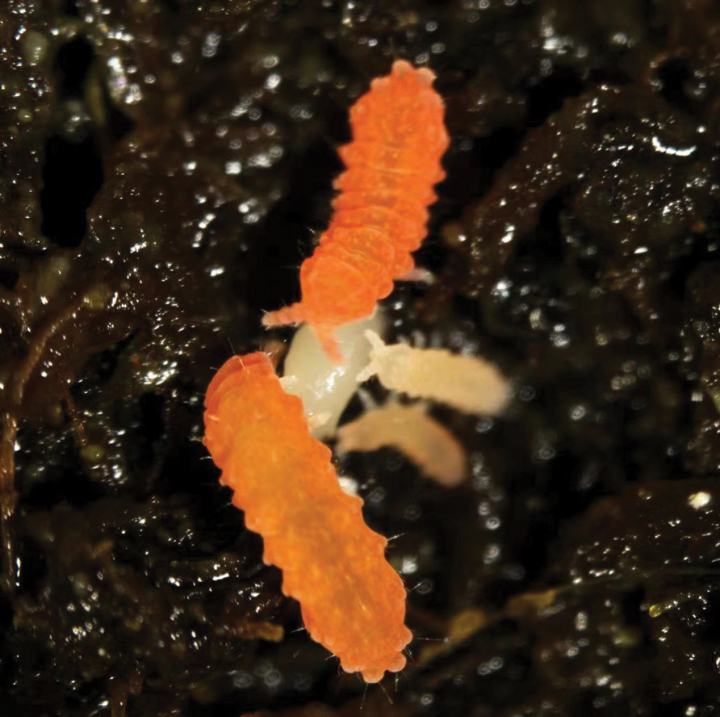
Live specimens of *Yuukianuraszeptyckii* feeding on yeast.

**Figure 16.**
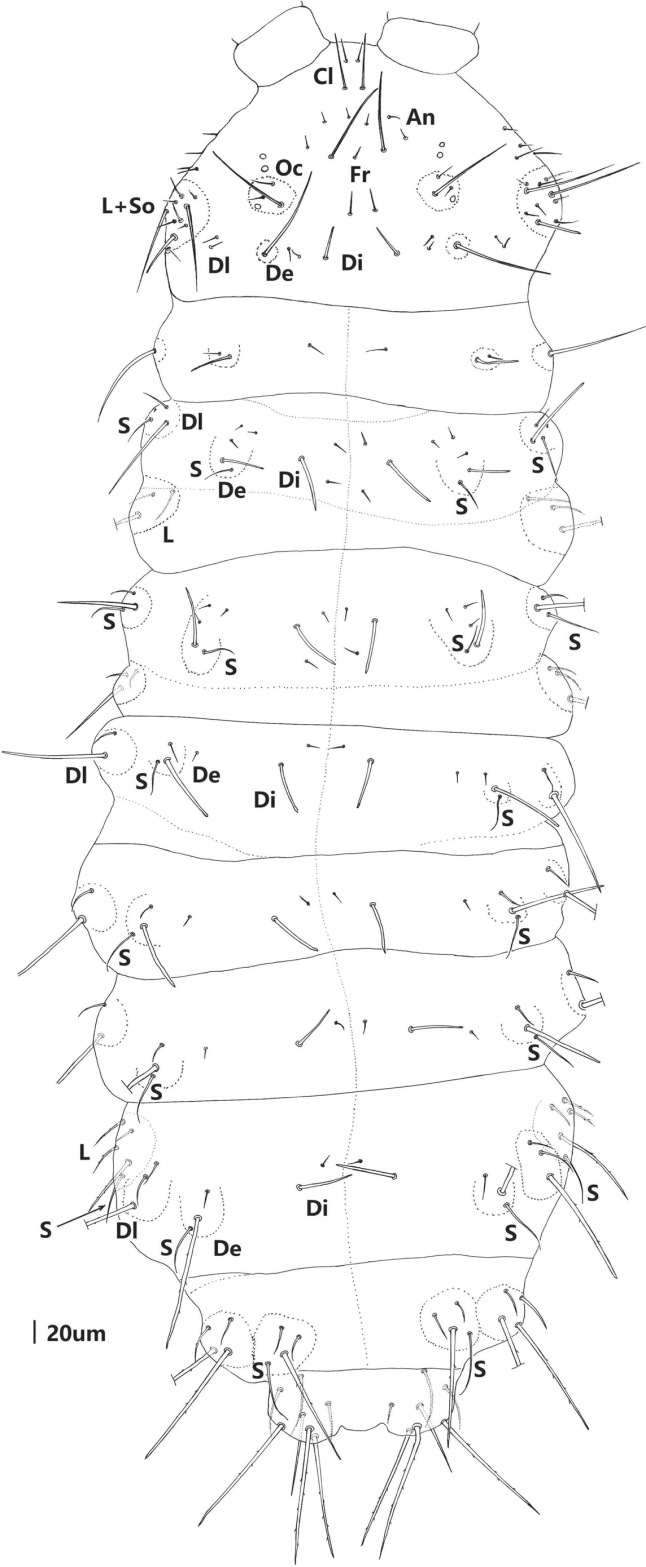
*Yuukianuraszeptyckii*: body dorsal tubercles and chaetotaxy.

**Figure 17.**
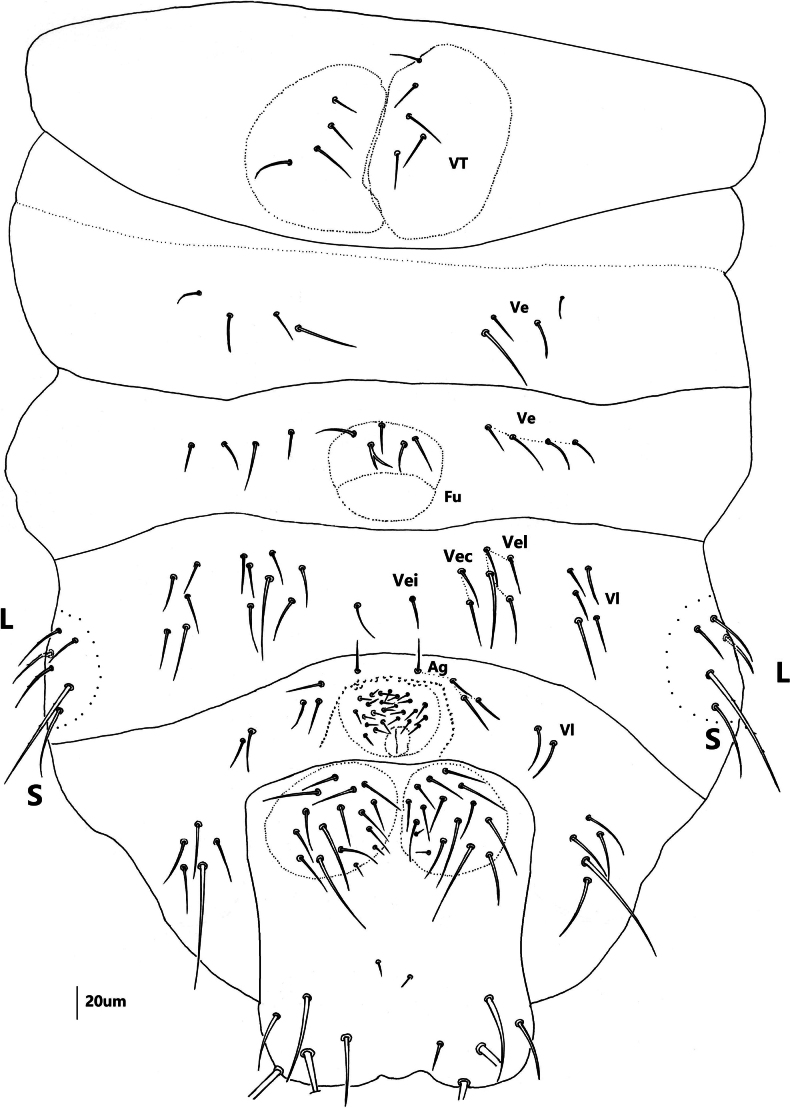
*Yuukianuraszeptyckii*: ventral side of Abd. I–VI.

**Figure 18.**
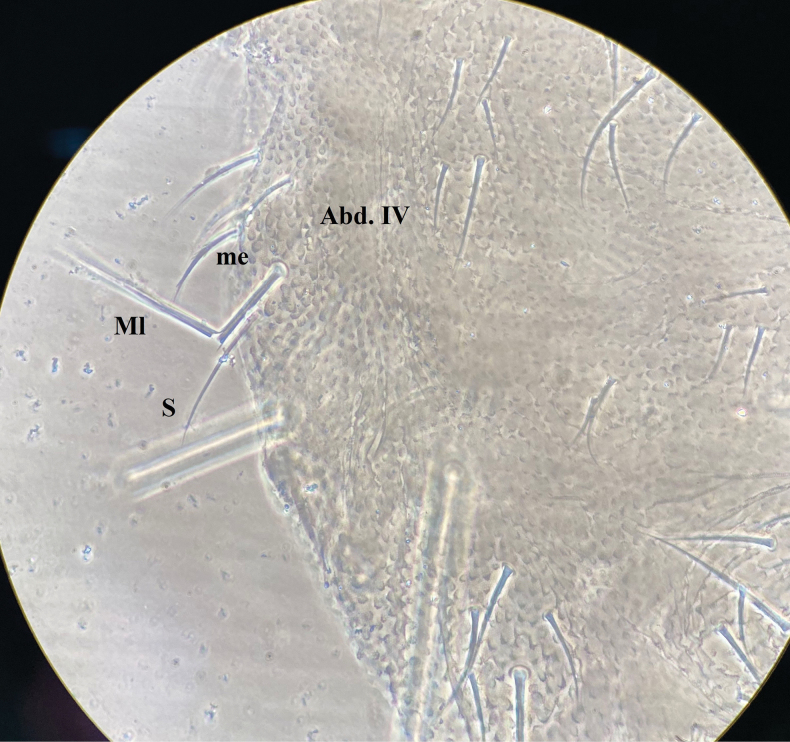
*Yuukianuraszeptyckii*: chaeta s on tubercle L of Abd. IV.

Figs 15 , 16 , 17 , 18 , Tables 3 , 4

###### Material.

• One male and two females, Pipa Lake, Nanjing, Jiangsu Province, China, coordinates: 32.0559°N, 118.8190°E, alt. ca 25 m, on bank of the lake, under a pile of aquatic grasses removed from the lake, leg. Daoyuan Yu.15.iv.2018. Materials are housed in the Key Laboratory of Zoology, Hunan University of Arts and Science (HUAS), Changde, Hunan Province, China.

###### Brief redescription.

Body length1.4–2.1 mm. Color. Reddish-orange while living (Fig. 15) and white in alcohol.

***Chaetal morphology*.** Dorsal ordinary chaetae of four types. Long macrochaetae (Ml) pointed or blunt, and serrated. Short macrochaetae (Mc) shorter than Ml, almost smooth. Short macrochaetae (Mcc) morphologically similar to and shorter than Mc. Mesochaetae (me) on body smooth or serrated, pointed and various lengths. Chaetae-s on terga thin, smooth, usually shorter than Mc, and longer than Mcc.

***Head*.** Eyes 3+3, uncolored, two eyes anterior to tubercle Oc, arrange one in front and one behind, the third one on posterior part of tubercle Oc (Fig. 16).

Buccal cone relatively short, labrum truncated, labral formula 0/2, 2.

Cephalic dorsal tubercles and chaetotaxy as in Fig. 16 and Table 3. Tubercles Oc, De and L+So poorly developed, tubercle Oc with 3 chaetae; De with 3 chaetae, Di1 and De1 as mes not on the tubercle; Dl with 2 chaetae; L and So fused to each other, with 2 Mls, 1 Mcc and 7 mes. Tubercle Fr with 3 chaetae, chaeta O far from chaeta A and close to An. Di with 1 Mcc. Cl, An, Fr and Di without tubercles.

**Table 3.** Cephalic tubercles and chaetotaxy of *Yuukianuraszeptyckii*.

Tubercle or group of chaetae	Tubercle	Number and type of chaetae	Names of chaetae
Cl	-	2 Mc	F
		2 me	G
Af	-	2 Ml	B
		2 Mcc	A
		7 me	C, D, E, O
Oc	+	1 Ml	Ocp
		2 me	Oca, Ocm
Di	-	1 Mcc	Di1 Di2
		1 me	
De	+	1 Ml	De1
		1 me	De2
Dl	-	2 me	Uncertain
L+So	+	2 Ml+1Mcc+8 me	Uncertain

Note, -: without distinct tubercle; +: with distinct tubercle.

***Thorax*** (Fig. 16 and Table 4). Thoracic tubercle Di not differentiated. Di on Th. I with 1 chaeta, Di on Th. II–III with 3 chaetae respectively. Tubercle De on Th. I–III faint but distinct, with 2, 4+S, 4+S chaetae respectively. Tubercle Dl of Th. I–III distinct, with 1, 2+S+ms, 2+S chaetae respectively. Tubercle L on Th. II–III feebly differentiated, with 3 chaetae each. Chaetotaxy of thorax and legs as in Table 1. Unguis with a basal inner tooth.

**Table 4.** Postcephalic dorsal tubercles and chaetotaxy of *Yuukianuraszeptyckii*.

Terga					Legs				
	Di	De	Dl	L	Scx2	Cx	Tr	Fe	T
Th. I	1	2	1	–	0	3	6	11	19
Th. II	3	4+s	2+s+ms	3	2	6	6	9	19
Th. III	3	4+s	2+s	3	2	8	6	9	18
Terga					Sterna				
Abd. I	2	3+s	2	3	VT: 4 (5)				
Abd. II	2	3+s	2	3	Ve: 3–4, Vel: 0				
Abd. III	2	3+s	2	3	Ve: 2–4, Fu: 4				
Abd. IV	2	2+s	3	4–5+s	Vei: 1, Vec: 2, Vel: 4–5, Vl: 5				
Abd. V	3+S+4			5	Ag: 3, Vl: 1, L’: 0				
Abd. VI	7				Ve: 15–16, An: 3 mi				

***Abdomen*** (Figs 16–18 and Table 4). Tubercle Di not differentiated on Abd. I–IV, and each with 2 chaetae. De poorly differentiated on Abd. I–IV, and shift laterally. De with 4 (3+s) chaetae on Abd. I–III respectively. Tubercle De on Abd. IV with 3 (2+s) chaetae. Tubercle Dl with 2 chaetae and L with 3 chaetae on Abd. I–III, both Dl and L poorly differentiated. Tubercle Dl on Abd. IV with 3 chaetae. Tubercle L on ventral Abd. IV always with 1 chaeta s, 1 Ml and 3–4 mes (Figs 16–18). Abd. V dorsally with 2+2 tubercles, tubercles Di and De completely fused, with 4(3+s) chaetae, tubercle Dl with 4 chaetae. However, tubercle Di+De partly fused to tubercle Dl. Abd. VI with one tubercle on each side, each with 7 chaetae. VT with 4 (sometimes 5) chaetae (Fig. 17). Furcular remnant with 5 chaetae (Fig. 17).

###### Ecology.

This species was found in humid litter of aquatic grasses piled on the shore of the lake.

###### Remarks.

*Yuukianuraszeptyckii* was first reported from North Korea, and later from Japan (Tanaka and Hasegawa 2010). Now it is for the first time reported from China, suggesting this species is probably widely distributed in East Asia.

So far, including the new species, a total of 11 *Yuukianura* species have been reported worldwide. Of these, five species—*Y.aphoruroides* (Yosii, 1953), *Y.deharvengi* Smolis, 2017, *Y.halophila* Yosii, 1955, *Y.rosea* (Kim & Lee, 2000), and *Y.szeptyckii*—have 3+3 eyes on the head. Among these five species, only *Y.deharvengi* has pigmented eyes. The remaining six species—*Y.hawaiiensis* (Bellinger & Christiansen, 1974), *Y.judithae* Deharveng, Palacios-Vargas & Bedos, 2017, *Y.kikaiensis* Kasai, 2024, *Y.pacifica* (Yosii, 1971), *Y.tongana* Yosii, 1964 and *Y.yasudai* (Yosii, 1966)—lack eyes entirely.

### ﻿Checklist of *Yuukianura* species and distributions

*Y.aphoruroides* (Yosii, 1953) Great Britain, China, Japan, Malaysia.

*Y.deharvengi* Smolis, 2017, Vietnam.

*Y.halophila* Yosii, 1955, Japan

*Y.hawaiiensis* (Bellinger & Christiansen, 1974), USA (Hawaii).

*Y.judithae* Deharveng, Palacios-Vargas & Bedos, 2017, Vanuatu.

*Y.kikaiensis* Kasai, 2024, Japan.

*Y.pacifica* (Yosii, 1971), Japan.

*Y.rosea* (Kim & Lee, 2000), South Korea.

*Y.szeptyckii* Deharveng & Weiner, 1984, North Korea, Japan, China (new record).

*Y.tongana* Yosii, 1964, The Kingdom of Tonga (Tonga islands).

*Y.yasudai* (Yosii, 1966), Nepal.

### ﻿Observation of the feeding behavior of *Yuukianuraszeptyckii* in culture

**Figure 19.**
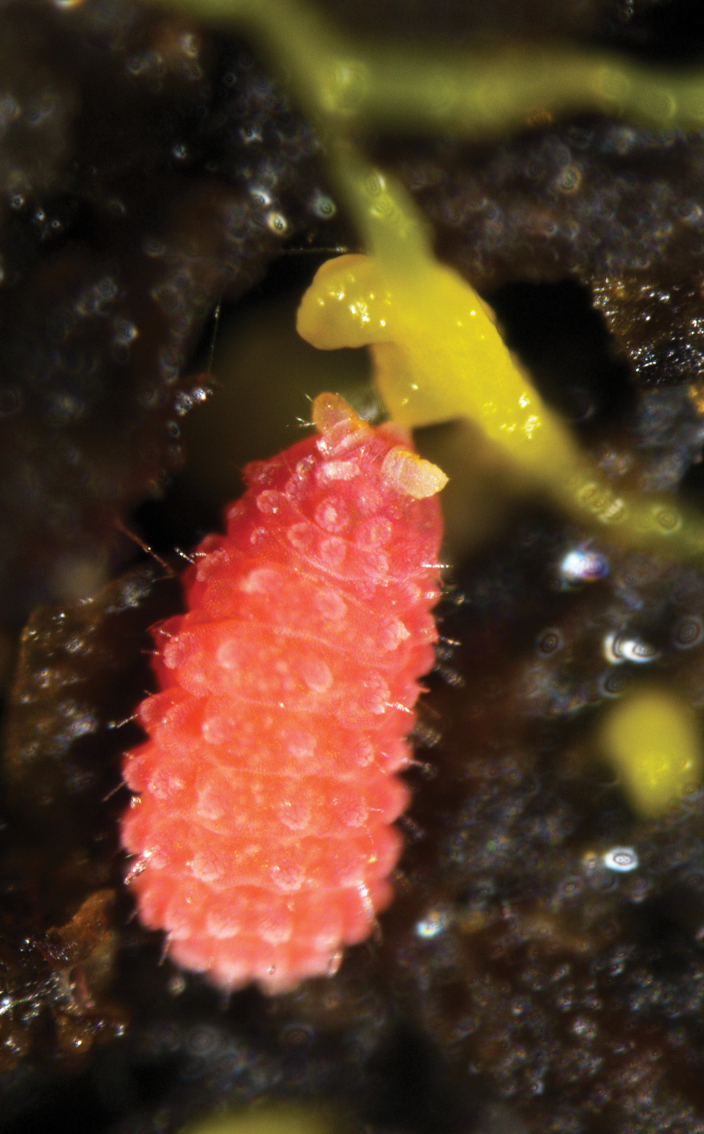
*Crossodonthina* sp. feeding on slime mold.

*Yuukianuraszeptyckii* showed equal feeding preferences for yeast and oats (Fig. 15), but did not feed on the slime mold. Under the feeding conditions of the first two food sources, *Y.szeptyckii* can successfully molt, lay eggs and hatch, thus completing its full life cycle (unpublished data). This feeding preference differs from that of other reported neanurids consuming slime molds (Fig. 19, also see Hoskins et al. 2015).

We also compared the feeding action of *Y.szeptyckii* with that of *Crossodonthina* sp. and *Orthonychiurus* sp. (Suppl. material 1). While it is feeding, *Y.szeptyckii* presses its buccal cone against the food surface and sticks out its maxillae to grasp food particles and brings them into its mouth, while its head keeps moving to find a suitable feeding spot (Suppl. material 1: Video Part A). This feeding action is similar to that of *Orthonychiurus* sp. (Suppl. material 1: Video Part B) and other families of Collembola, but differs from that of *Crossodonthina* sp. of the same family (same subfamily and tribe). *Crossodonthina* sp. (which feeds only on slime molds among the three food resources provided) inserts the tip of its buccal cone into the plasmodia and veins of the slime mold to suck out protoplasm, while keeping its head and body still, which is similar to the feeding action reported in other species of Neanuridae (Hoskins et al. 2015). Mouthpart movements are detectable only by subtle head tremors (Suppl. material 1: Video Part C).

In summary, the feeding preference and action of *Y.szeptyckii* differ significantly from *Crossodonthina* sp., while resembling those of non-neanurid Collembola, such as *Orthonychiurus*. This suggests that this group (*Yuukianura*) may occupy a unique position within the family Neanuridae. Intriguingly, both groups we have observed, namely *Yuukianura* and *Crossodonthina*, have more complicated mouthparts among Neanurinae, but only the former showed different feeding behavior from others (sensu Hoskins et al. 2015), suggesting a potential mismatch between morphology and function. Future research should focus on observing the feeding behavior of other *Yuukianura* species and other genera of Neanuridae, in combination with molecular analyses, to determine the phylogenetic position of this genus, evaluate the relatedness of feeding behavior to phylogeny, and reveal the exact correlation between the morphologies of mouthparts and their functions.

## Supplementary Material

XML Treatment for Vitronura cheni

XML Treatment for Yuukianura szeptyckii

## ﻿Additional information

### Use of AI

No use of AI was reported.

### Author ORCIDs

Ji-Gang Jiang https://orcid.org/0000-0002-4484-3312

Daoyuan Yu https://orcid.org/0000-0003-2984-0540

### Data availability

All of the data that support the findings of this study are available in the main text or Supplementary Information.

## Supplementary materials

Supplementary material 1 Supplementary videos Data type mp4 Explanation note Supplementary videos on YouTube (https://youtu.be/_SVKQAGRTvw): **A***Yuukianura* feeding on yeast **B***Orthonychiurus* feeding on yeast **C***Crossodonthina* feeding on slime mold. https://binary.pensoft.net/file/1374374This dataset is made available under the Open Database License (http://opendatacommons.org/licenses/odbl/1.0/). The Open Database License (ODbL) is a license agreement intended to allow users to freely share, modify, and use this Dataset while maintaining this same freedom for others, provided that the original source and author(s) are credited.Ji-Gang Jiang, Daoyuan Yu
